# Barrier Nursing in Fever Cases

**Published:** 1921-08-13

**Authors:** 


### Barrier Nursing in Fever Cases.

On Tuesday, July 26, in the Lecture Theatre of the General Hospital, Birmingham (by kind permission of the Governors), Dr. Harries, Medical Superintendent of the City Hospital, Little Bromwich, gave an interesting lecture to members of the Birmingham and Three Counties Centre of the College Local Centre on "Barrier Nursing in Fever Cases." Dr. Harries declared that the success of barrier nursing can only be attained if the ward sister and staff nurses are general-trained and proficient in surgical asepsis. The technique of barrier nursing is based on the fact that infectious diseases are spread not by infection through air, but by immediate or intermediate contact; smallpox is the exception to this rule. The first experiment in barrier nursing took place in Paris twenty years ago, when glass screens were erected between the beds and the cubicle system was introduced. This was superseded by the abolition of the screens and the introduction of the invisible barrier, the beds being marked by a blue band for definite fevers and a red band for observation or questionable cases. In Liverpool the distinctive marks have now been abolished, and the Medical Superintendent relies solely on the skill and efficiency of the nurses.

Dr. Harries detailed the advantages of barrier nursing as follows: During an epidemic, when small isolation wards are not obtainable, suspicious rashes can be kept under observation; patients suffering from one definite fever who have been in contact with relatives suffering from another can be isolated; patients recovering from one fever, having contracted a second, can be admitted without endangering others; patients can be warded for observation.

The disadvantages are: The heavy strain entailed on the nurses; the large nursing *personnel* required—a ward of thirty-two beds needs one sister, two staff nurses (general-trained), and eight probationers; the amount of floor space: the authorised space between the beds is 12 ft., the space from foot to foot 16½ ft.

At the conclusion of his lecture Dr. Harries cordially invited the nurses present to visit the City Hospital and see barrier nursing in practice.

